# Whole Genome Sequencing Demonstrates Limited Transmission within Identified *Mycobacterium tuberculosis* Clusters in New South Wales, Australia

**DOI:** 10.1371/journal.pone.0163612

**Published:** 2016-10-13

**Authors:** Ulziijargal Gurjav, Alexander C. Outhred, Peter Jelfs, Nadine McCallum, Qinning Wang, Grant A. Hill-Cawthorne, Ben J. Marais, Vitali Sintchenko

**Affiliations:** 1 Marie Bashir Institute for Infectious Diseases and Biosecurity, The University of Sydney, Sydney, Australia; 2 Centre for Infectious Diseases and Microbiology–Public Health, Westmead Hospital, Sydney, Australia; 3 Children's Hospital at Westmead, Sydney, Australia; 4 NSW Mycobacterium Reference Laboratory, Centre for Infectious Diseases and Microbiology Laboratory Services, Institute of Clinical Pathology and Medical Research–Pathology West, Sydney, Australia; 5 School of Public Health and Westmead Institute for Medical Research, The University of Sydney, Sydney, Australia; University of Minnesota, UNITED STATES

## Abstract

Australia has a low tuberculosis incidence rate with most cases occurring among recent immigrants. Given suboptimal cluster resolution achieved with 24-locus mycobacterium interspersed repetitive unit (MIRU-24) genotyping, the added value of whole genome sequencing was explored. MIRU-24 profiles of all *Mycobacterium tuberculosis* culture-confirmed tuberculosis cases diagnosed between 2009 and 2013 in New South Wales (NSW), Australia, were examined and clusters identified. The relatedness of cases within the largest MIRU-24 clusters was assessed using whole genome sequencing and phylogenetic analyses. Of 1841 culture-confirmed TB cases, 91.9% (1692/1841) had complete demographic and genotyping data. East-African Indian (474; 28.0%) and Beijing (470; 27.8%) lineage strains predominated. The overall rate of MIRU-24 clustering was 20.1% (340/1692) and was highest among Beijing lineage strains (35.7%; 168/470). One Beijing and three East-African Indian (EAI) clonal complexes were responsible for the majority of observed clusters. Whole genome sequencing of the 4 largest clusters (30 isolates) demonstrated diverse single nucleotide polymorphisms (SNPs) within identified clusters. All sequenced EAI strains and 70% of Beijing lineage strains clustered by MIRU-24 typing demonstrated distinct SNP profiles. The superior resolution provided by whole genome sequencing demonstrated limited *M*. *tuberculosis* transmission within NSW, even within identified MIRU-24 clusters. Routine whole genome sequencing could provide valuable public health guidance in low burden settings.

## Introduction

*Mycobacterium tuberculosis* is a highly successful human pathogen. Estimates suggest that up to a third of global people are infected and that 9 million developed active disease in 2014 [1]. Recent data support the ancient origins of *M*. *tuberculosis* with evidence of ongoing adaptation and genetic diversification throughout human history [2]. Seven *M*. *tuberculosis* strain lineages have been identified with distinct geographic distribution patterns, shaped by ancient human migration pathways [2,3]. More recent migration patterns and increased population mobility are reshaping these geographic distributions.

Traditional genotyping methods such as mycobacterial interspersed repetitive unit (MIRU) analysis provided new insight into pathogen diversity and strain-specific transmission dynamics [4]. Different *M*. *tuberculosis* strain lineages have been associated with variable virulence, transmissibility, disease phenotypes and drug resistance profiles [5–7]. Molecular epidemiology studies also confirmed the transmissibility of drug-resistant strains and highlighted the importance of re-infection in tuberculosis (TB) endemic settings with uncontrolled transmission [8,9]. More recently the availability of whole-genome sequencing (WGS) has provided unprecedented strain resolution to enhance our understanding of *M*. *tuberculosis* evolution and transmission [10,11].

Australia is a low TB incidence setting with a stable incidence of 5–6 per 100,000 [12]. The most populous state of Australia, New South Wales (NSW), with 7.4 million population reports the highest TB case numbers with significant geographic clustering in and around Sydney [13]. The vast majority of TB cases occur among immigrants from high TB incidence countries [14]. Analysis of routine MIRU typing data showed that East African Indian (EAI) and Beijing lineage strains were most common, with a recent increase in the relative abundance of EAI lineage strains related to changes in migrant flows [4]. It also demonstrated high strain diversity within identified geographic hotspots, suggesting foci of imported disease rather than clusters of local transmission [15].

Despite these insights, the existence of clusters with identical MIRU-24 profiles provided a public health dilemma, since genotypic clustering is suggestive of local transmission that may require targeted public health intervention [4]. It is well recognized that accurate cluster identification is problematic with strains that are highly monomorphic and poorly differentiated using MIRU, such as Beijing lineage strains [16]. In order to assess the contribution of local TB transmission and guide public health intervention efforts we performed WGS of identified MIRU clusters to establish the frequency of true transmission chains within these clusters.

## Materials and Methods

### Genotyping of bacterial strains

Routine surveillance data collected between 2009 and 2013 at the Mycobacterium Reference Laboratory, NSW containing all culture confirmed *M*. *tuberculosis* isolates 24-loci MIRU (MIRU-24) genotypes and demographic data were reviewed. MIRU-24 genotyping was performed as described earlier [4] and used to (a) assign strain lineage using the miru-vntrplus.org online database, (b) to build a minimum spanning tree using Bionumerics v.5 (Applied-Maths, Kortrijk, Belgium), (c) to calculate allelic richness (AR) using HP-RARE software v.1 [17] and finally (d) to calculate Hunter Gaston Index of Diversity (HGDI, with the assumption that isolates were unrelated) [18]. In addition, 12-locus MIRU was employed to identify EAI sublineages in the SITVIT v.2 database [19]. Two or more isolates sharing an identical MIRU-24 profile were considered a genotype cluster and aggregated on the minimum spanning tree as a node. Isolates with a difference of one or more MIRU-24 loci were represented by separate nodes; the distance between respective nodes (indicated by the number of line segments) reflected the number of MIRU-24 loci differences. The “rate of recent transmission” was calculated as follows [(number of clustered isolates—number of clusters) x 100/total number of cultured isolates] [4].

### Whole-genome sequencing

The largest four MIRU-24 clusters identified during the study period were selected for WGS. These 4 clusters belonged to EAI Lineage 1 (clusters A and B with 5 and 4 isolates each) and Beijing Lineage 2 (clusters C and D with 11 and 10 isolates each) strains. Selected cultures of *M*. *tuberculosis* were initially stored at -80°C and then recovered on Middlebrook agar. Genomic DNA was extracted using Wizard Genomic DNA purification kit (Promega, Madison, WI, USA) and libraries were prepared using Ion Xpress Plus Fragment Library kit (Life Technologies, Gaithersburg, MD, USA). Sequencing was performed on an Ion Torrent Personal Genome Machine (Agilent Technologies, Palo Alto, CA, USA) with the Ion 318 chip kit (Life Technologies) as per manufacturer’s instructions. A reference genome for mapping was prepared from NC_000962.3 [20] by substituting gaps in place of repetitive elements (all regions annotated as PE/PPE/PGRS and *cysA* genes, insertion sequences, transposases and prophage components, with gaps representing 6.3% of NC_000962.3.RRE). Single nucleotide polymorphisms (SNPs) were called using the *mem* algorithm of the mapper *bwa* [21], followed by the variant-caller *freebayes* [22] operating on merged lineage-specific BAM files, with “ODDS > 99” used as a quality filter (“ODDS” is a composite value that represents marginal likelihood [23]; the resulting VCF files can be found in the S1 File). Lineage-specific whole-genome Bayesian inference substitution trees were generated with *mrbayes* [24], using NC_000962.3.RRE patched with SNPs (all non-SNP variants were excluded by filtering for CIGAR1X). A synthetic lineage-specific most recent common ancestor (MRCA) was generated by patching NC_000962.3.RRE with all SNPs shared within that lineage, and included in each tree to serve as a comparator. A difference between libraries of less than 10 SNPs was considered a SNP cluster, suggestive of recent transmission [24].

### Statistical analysis and ethics approval

Demographic and clinical characteristics of different *M*. *tuberculosis* lineages were explored using descriptive statistics, using χ^2^ and One-way ANOVA tests where applicable. All statistical analyses were performed using SPSS 23.0 (IBM, USA) and p-values less than 0.05 were considered significant. The study was approved by the Human Research Ethics Committee of the University of Sydney (project number 2013/126).

## Results

A total of 1841 culture-confirmed TB cases were identified; 72% of all TB cases in NSW during the study period. Of these, 1692 (91.9%) had complete demographic and MIRU-24 genotyping data. The *M*. *tuberculosis* population structure included 17 different strain families, with Lineage 1 (EAI, 28%, n = 474) and Lineage 2 (Beijing, 27.8%, n = 470) accounting for more than half of all strains identified. Demographic, clinical and MIRU-24 clustering characteristics of the predominant strain families compared to minority strains are presented in Table 1. The mean age of patients infected with Beijing and EAI lineage strains was similar (43 and 45 years respectively, p = 0.02), but Beijing strains caused disease with a bi-phasic age distribution being most common in young adults (15-29yrs) and in older people (>60yrs). Compared to all other cases, Beijing lineage strains were strongly associated with multi-drug resistant TB (odds ratio 5.0, CI 95% 1.7–14.9, p = 0.04).

**Table 1:** Demographic, clinical and MIRU-24 clustering characteristics of predominant *M*. *tuberculosis* strain lineages.

Characteristics		EAI^#^	Beijing	Other^##^	Total	p value
		n (%)	n (%)	n (%)	n (%)	
		N = 474	N = 470	N = 748	N = 1692	
**Age group**						
	<15	6 (1.3)	2 (0.4)	14 (1.9)	22 (1.3)	0.001
	15–29	101 (21.3)	197 (41.9)	278 (37.2)	576 (34.0)	
	30–44	150 (31.6)	79 (16.8)	185 (24.7)	414 (24.5)	
	45–59	113 (23.8)	75 (16.0)	110 (14.7)	298 (17.6)	
	≥60	104 (21.9)	117 (24.9)	161 (21.5)	382 (22.6)	
**Gender**						
	Male	272 (57.4)	264 (56.2)	437 (58.4)	973 (57.5)	0.74
**Site of infection**						
	Respiratory	291 (61.4)	347 (73.8)	518 (69.3)	115 (68.3)	0.001
**Drug resistance**						
** **	Isoniazid (low and high level)	40 (8.4)	51 (10.9)	46 (6.1)	137 (8.1)	0.001
** **	MDR/XDR*	4 (0.8)	19 (4.0)	11 (1.5)	34 (2.0)	
**MIRU-24 clustering**						
** **	No of clusters	32	54	37	123	
** **	No of clustered isolates (%)	76 (16.0)	168 (35.7)	96 (12.7)	340 (20.1)	
	Average cluster size (range)	2 (2–5)	3 (2–11)	2 (2–8)	2 (2–12)	
	Estimated transmission rate^&^	9.3	24.3	7.9	12.8	

^#^EAI–East African Indian lineage strains

^##^ Other–minority strain lineages pooled together

*MDR/XDR–multi-drug resistant tuberculosis (including a single case of extensively drug resistant tuberculosis diagnosed in 2011); MIRU-24–24-locus mycobacterium interspersed repetitive unit strain typing method

^&^Estimated transmission rate = [(number of clustered isolates—number of clusters)/ total number of genotyped isolates]

**Fig 1:**
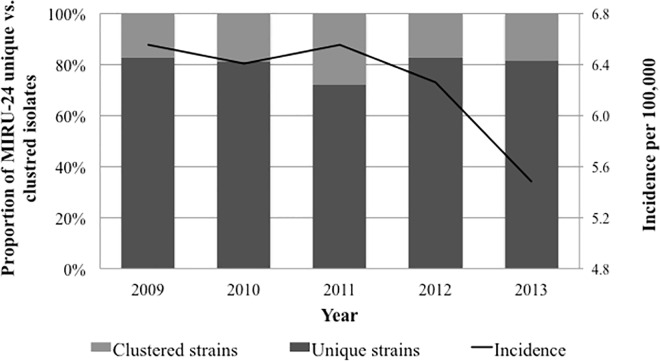
Tuberculosis incidence and genotypic clustering rate in NSW, Australia. MIRU-24–24-locus mycobacterium interspersed repetitive unit strain typing method.

The TB incidence in NSW showed a decrease from 6.5/100,000 population in 2009 to 5.5/100,000 population in 2013 (Fig 1). Apart from 2011, when nearly a quarter of strains were clustered by MIRU-24, less than 20% of strains were clustered during each of the study years. The average clustering rate of 20.1% (340/1692) suggested that up to 12.8% of cases may have resulted from recent transmission, although the calculated mean cluster sizes were small (2–3 cases). Beijing family strains demonstrated the highest degree of clustering (168/470; 35.7%); comprising 49.4% (168/340) of all clustered stains (Table 1 and S1 Fig). The estimated recent transmission rate was highest among Beijing strains (24.3%), with cluster sizes varying between 2 and 11 cases (Table 1).

**Fig 2:**
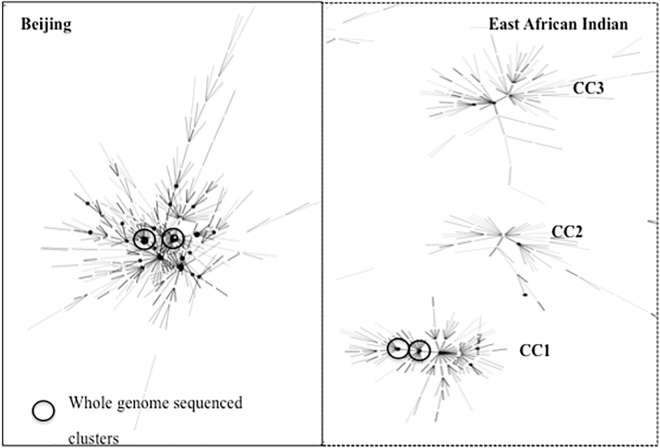
MIRU-24 minimum spanning tree of the predominant *M*. *tuberculosis* lineages identified in NSW, Australia. MIRU-24–24-locus mycobacterium interspersed repetitive unit strain typing method; Solid box shows single large clonal complex for Beijing; Dotted box shows 3 independent clonal complexes of East African Indian strain lineage; CC1 –clonal complex 1; CC2 –clonal complex 2; CC3 –clonal complex 3; Circles indicate clusters that were subjected to whole genome sequencing.

MIRU-24 based minimum spanning tree analysis of Beijing lineage strains identified a single clonal complex with four large nodes, while three independent complexes, each with 1–2 large nodes, were observed among EAI lineage strains (Fig 2). The HGDI index for EAI strains (0.43) was slightly higher than for Beijing lineage strains (0.25), as was the mean allelic richness (3.6 vs. 2.7; p = 0.065); MIRU loci 1955 and 2163 displayed the highest variability (S2 Fig). The nodes identified within the three EAI clonal complexes belonged to the following MIRU international types (MIT): complex 1 MIT56; complex 2 MIT59 and MIT272; and complex 3 MIT69 and MIT409 (S3 Fig).

All generated raw reads from whole-genome sequencing were submitted to the European Nucleotide Archive of the European Bioinformatics Institute under study accession number PRJEB11778. The ENA sample identification numbers are listed in S1 Table. After mapping, median read depth ranged from 40- to 100-fold, with a single library at 22-fold depth; for each library, reads covered >91% of the reference genome, and >91% of reads were mapped to the reference genome. VCF files listing the variants found (including filtered, informative SNPs annotated with snpEff [25]) are including in the S1 File. Resistance-associated SNPs that could have contributed to homoplasy (affecting subsequent phylogenetic inference) were sought but not found.

**Fig 3:**
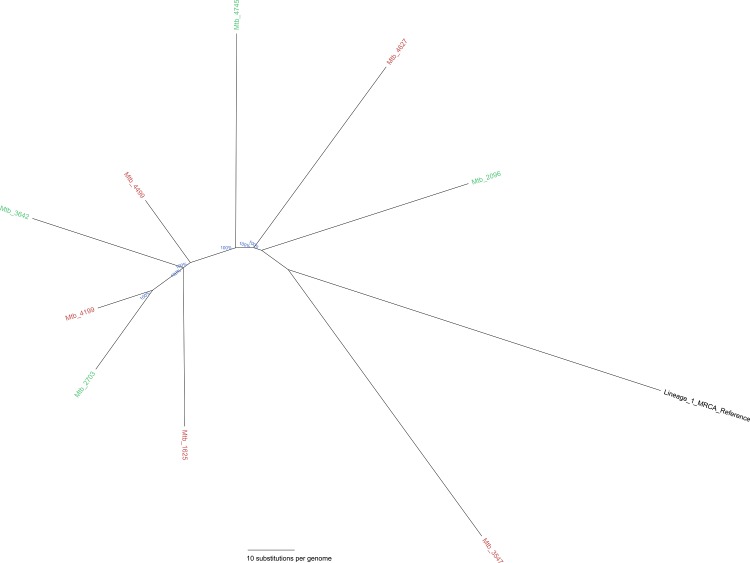
Whole-genome Bayesian inference distance tree of Lineage 1 East African Indian (EAI) clusters with identical MIRU-24 profiles (MIRU-24 clusters). MIRU-24 cluster A is labeled in red, and MIRU-24 cluster B in green and were only distantly related (>100 SNP differences) on whole genome sequencing without any SNP clusters identified.

**Fig 4:**
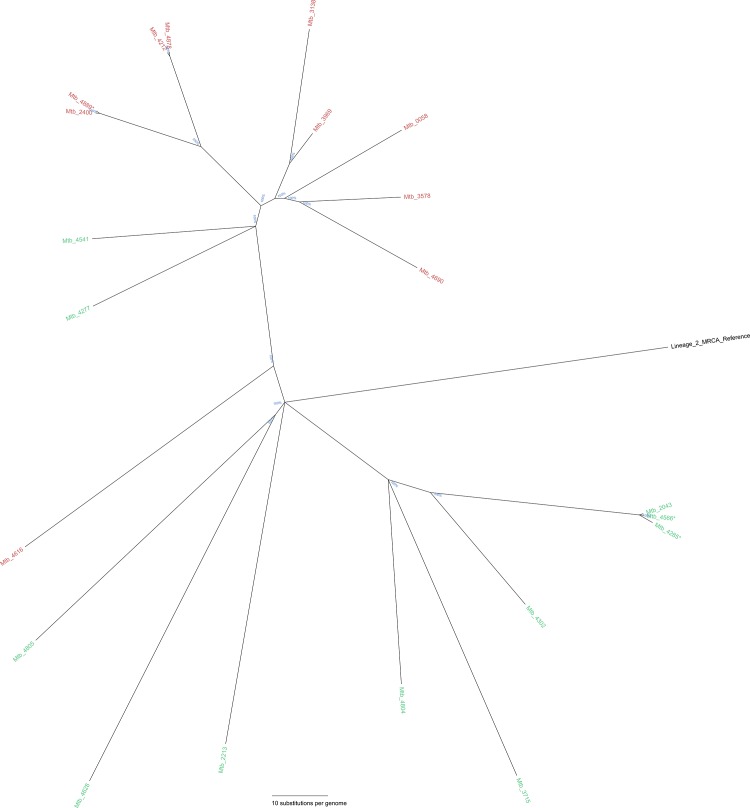
Whole-genome Bayesian inference distance tree of Lineage 2 Beijing clusters with identical MIRU-24 profiles (MIRU-24 clusters). MIRU-24 cluster C is labeled in green, and MIRU cluster D in red and three SNP clusters were identified. Three libraries of the two SNP clusters were determined to represent cross-contamination during diagnostic culture are marked with an asterisk. Branch support probabilities are displayed as percentages in blue. The scale bars represents 10 substitutions per genome for the corresponding distance from a node. Parameters, output and version information for *mrbayes* can be found in S2 File.

Fig 3 reflects the whole-genome Bayesian inference substitution tree for Lineage 1 MIRU-24 clusters A and B (identical MIRU-24 profiles); both from clonal complex1. WGS demonstrated no SNP clusters within MIRU-24 clusters A and B, thereby reducing the MIRU-based calculated transmission rate by 100%. Demographic and epidemiological data demonstrated no link between these cases, suggesting that strains with identical MIRU-24 profiles were acquired overseas; 7 in the Philippines, one in Ethiopia and the country of origin remained unknown for one patient. SNP-based phylogenetic analysis of clusters C and D from within the Lineage 2 clonal complex identified by MIRU-24 (Fig 4) demonstrated 3 small SNP clusters embedded within. MIRU-24 cluster C included a single 3-member SNP cluster (0–2 SNP differences). Importantly, two of three isolates from this SNP cluster were classified as probable laboratory cross-contamination following careful assessment by laboratory and public health investigators. MIRU-24 cluster D contained two SNP clusters with two members each (0 SNP differences); one of these clusters contained another isolate that was classified as probable laboratory contamination by laboratory and public health investigators. In hindsight, of the 21 Beijing/Lineage 2 isolates clustered by MIRU-24, only a single 2-member SNP cluster represented likely transmission.

## Discussion

WGS demonstrated that large *M*. *tuberculosis* genotype clusters identified with routine MIRU-24 typing were not indicative of local transmission in this low TB incidence setting dominated by imported disease. The use of WGS-defined SNP clusters reduced the MIRU-24 potential secondary case rate for Beijing lineage strains by 79% (from 19/19 to 4/19) with a further 16% reduction (from 4/19 to 1/19) once likely laboratory contamination events were excluded. Overall SNP-based analysis of the four large MIRU-24 clusters (30 specimens in total) suggested only a single case of local transmission (reducing the number of potential secondary cases from 26 to 1). The fact that laboratory contaminated isolates were clustered by WGS allowed for a critical review of specimen collection and processing methods and offered practical guidance to public health authorities. We reconfirmed previous observations [26] that Beijing lineage strains are most prevalent among young adults, with increased rates of respiratory disease and drug resistance compared to other strains. Our findings highlighted the challenge of interpreting MIRU-24 clusters for highly homoplastic Beijing lineage strains [27], particularly in low incidence settings dominated by imported disease.

Interestingly, WGS also demonstrated added value in the evaluation of EAI lineage clusters, despite higher MIRU-24 allelic diversity observed in these strains. Not a single transmission chain was identified within the largest MIRU-24 clusters selected (9 isolates in total). The observation that EAI lineage strains were more prevalent than Beijing among patients with non-respiratory TB and demonstrated equal spread across the age spectrum, support previous observations that suggested reduced transmissibility of EAI strains [28,29]. The EAI MIRU-24 clonal complexed identified are well recognized within the Asia-Pacific region and may provide a clue as to the likely geographic origin of these strains. For example MIT56 identified within clonal complex 1 has been found in the Philippines and Japan [30,31], while MIT59 and MIT272 identified within clonal complex 2 is known to circulate in Vietnam [32,33]. Together these findings shed important light on the likely routes of importation of various *M*. *tuberculosis* strains into Australia, which may influence pre- and post-immigration screening practices [15] and direct Australian support for TB control initiatives in the region [34].

The sub-optimal resolution associated with MIRU-24 genotyping could be attributable to a slow molecular clock (depending on the experiment, it ranges from 1x10^-5^ to 1x10^-2^ per locus per year) and the fact that it considers less than 1% of the *M*. *tuberculosis* genome, compared to an estimated SNP mutation rate of 0.3 SNP per genome per year [20,35] with an assessment of the majority of genome; excluding only PE/PPE and PGRS family genes from the genomic comparison. The lower MIRU-24 allelic diversity and single large MIRU-24 cluster of Beijing lineage strains identified are not unexpected, given that Beijing lineage strains have less genetic diversity than EAI lineage strains [36]. Interestingly, despite its reduced MIRU-24 allelic diversity (Table 1), Beijing lineage strains have been associated with increased rates of drug resistance. However, the mechanisms underlying the generation and spread of drug resistant mutations are completely different to those generating MIRU-24 allelic diversity.

Study limitations include the fact that we sequenced only 4 large genotype clusters, two each from Beijing and EAI lineage strains. These two lineages accounted for 55.8% of all culture-confirmed TB cases in NSW and makes up 71.8% of all clustered cases. Although this limited WGS analysis does not represent all transmission events in NSW, it does indicate that false MIRU-24 clustering is a major problem in low incidence settings and that local transmission is less common than MIRU-24 clustering suggests. A recent study from the United Kingdom demonstrated that routine WGS is poised to replace conventional methods in mycobacterial reference laboratories, given the rapidly reducing cost as well as clinical and public health relevance of the findings [37]. Apart from improving the accuracy of assessing recent transmission events, WGS may reduce costs related to unnecessary contact investigations in low incidence settings [38].

In conclusion, MIRU-24 typing could lead to false cluster identification, especially with highly monomorphic *M*. *tuberculosis* strains lineages, such as Beijing. This is a particular problem in low burden settings with minimal local TB transmission, where most TB cases represent imported disease. Application of WGS to assess SNP clusters improves the accuracy of recent transmission estimates and provides valuable public health guidance. Thus we suggest that routine WGS should replace traditional genotyping methods in low burden settings with adequate resources.

## Supporting Information

S1 Table The European Nucleotide Archive sample identification numbers. (DOCX)

S1 Fig *M*. *tuberculosis* strain lineage distribution among genotypically clustered and unique strains. (PPTX)

S2 Fig Comparison of MIRU-24 loci allelic richness for EAI and Beijing strain lineages. (PPTX)

S3 Fig Minimum spanning tree of all culture-confirmed and MIRU-24 typed *M*. *tuberculosis* isolates identified between 2009–2013 in NSW, Australia. (PPTX)

S1 File VCF files describing the variants found using whole-genome sequencing. (ZIP)

S2 File Settings, output and version information for mrbayes. (ZIP)
