# Supplementary material for: Whole Genome Sequencing Demonstrates Limited Transmission within Identified Mycobacterium tuberculosis Clusters in New South Wales, Australia
Source: PLoS One. 2016 Oct 13;11(10):e0163612. doi: 10.1371/journal.pone.0163612 (PMC5063377; doi:10.1371/journal.pone.0163612)
Supplement: S1 Table — (DOCX) [file pone.0163612.s001.docx]

**Supplementary Materials**

**S1 Table.** The European Nucleotide Archive sample identification numbers

Study accession number PRJEB11778 (<http://www.ebi.ac.uk/ena/data/view/PRJEB11778>).

Sample accession        Secondary accession     Sample unique name
ERS979051       SAMEA3671902    Mtb_0058
ERS979052       SAMEA3671903    Mtb_2400
ERS979053       SAMEA3671904    Mtb_3578
ERS979054       SAMEA3671905    Mtb_4212
ERS979055       SAMEA3671906    Mtb_4616
ERS979056       SAMEA3671907    Mtb_4690
ERS979057       SAMEA3671908    Mtb_4878
ERS979058       SAMEA3671909    Mtb_4889
ERS979059       SAMEA3671910    Mtb_3138
ERS979060       SAMEA3671911    Mtb_2043
ERS979061       SAMEA3671912    Mtb_2213
ERS979062       SAMEA3671913    Mtb_3715
ERS979063       SAMEA3671914    Mtb_4277
ERS979064       SAMEA3671915    Mtb_4285
ERS979065       SAMEA3671916    Mtb_4302
ERS979066       SAMEA3671917    Mtb_4541
ERS979067       SAMEA3671918    Mtb_4566
ERS979068       SAMEA3671919    Mtb_4628
ERS979069       SAMEA3671920    Mtb_4804
ERS979070       SAMEA3671921    Mtb_4805
ERS979071       SAMEA3671922    Mtb_2096
ERS979072       SAMEA3671923    Mtb_2703
ERS979073       SAMEA3671924    Mtb_3642
ERS979074       SAMEA3671925    Mtb_4745
ERS979075       SAMEA3671926    Mtb_3547
ERS979076       SAMEA3671927    Mtb_4199
ERS979077       SAMEA3671928    Mtb_4627
ERS979078       SAMEA3671929    Mtb_4499
ERS979079       SAMEA3671930    Mtb_1625
