# Supplementary material for: Whole Genome Sequencing Demonstrates Limited Transmission within Identified Mycobacterium tuberculosis Clusters in New South Wales, Australia
Source: PLoS One. 2016 Oct 13;11(10):e0163612. doi: 10.1371/journal.pone.0163612 (PMC5063377; doi:10.1371/journal.pone.0163612)
Supplement: S1 Fig — (PPTX) [file pone.0163612.s002.pptx]

## Slide 1
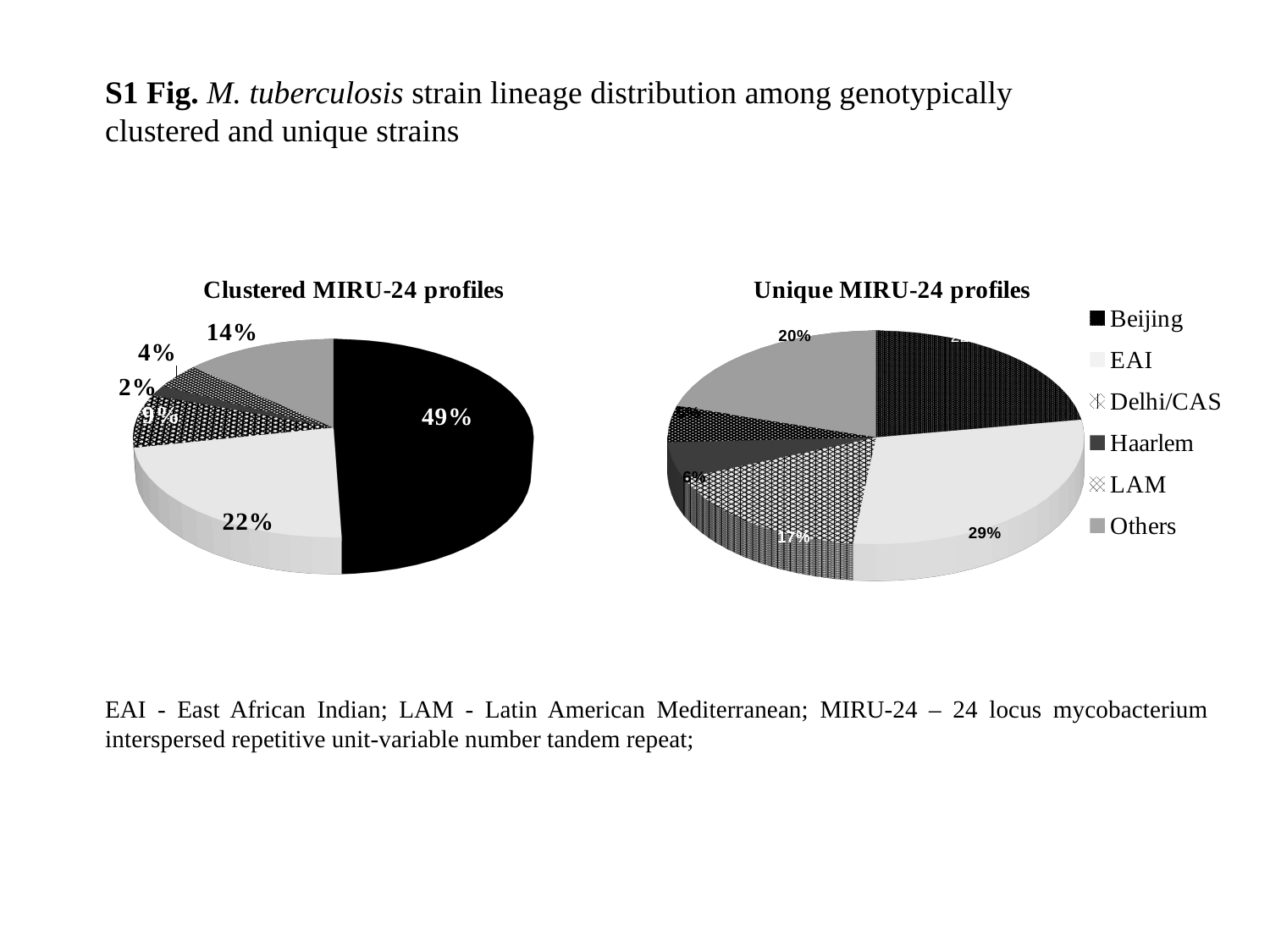

S1 Fig. M. tuberculosis strain lineage distribution among genotypically clustered and unique strains
[unsupported chart]
[unsupported chart]
EAI - East African Indian; LAM - Latin American Mediterranean; MIRU-24 – 24 locus mycobacterium interspersed repetitive unit-variable number tandem repeat;
