# Supplementary material for: Whole Genome Sequencing Demonstrates Limited Transmission within Identified Mycobacterium tuberculosis Clusters in New South Wales, Australia
Source: PLoS One. 2016 Oct 13;11(10):e0163612. doi: 10.1371/journal.pone.0163612 (PMC5063377; doi:10.1371/journal.pone.0163612)
Supplement: S2 Fig — (PPTX) [file pone.0163612.s003.pptx]

## Slide 1
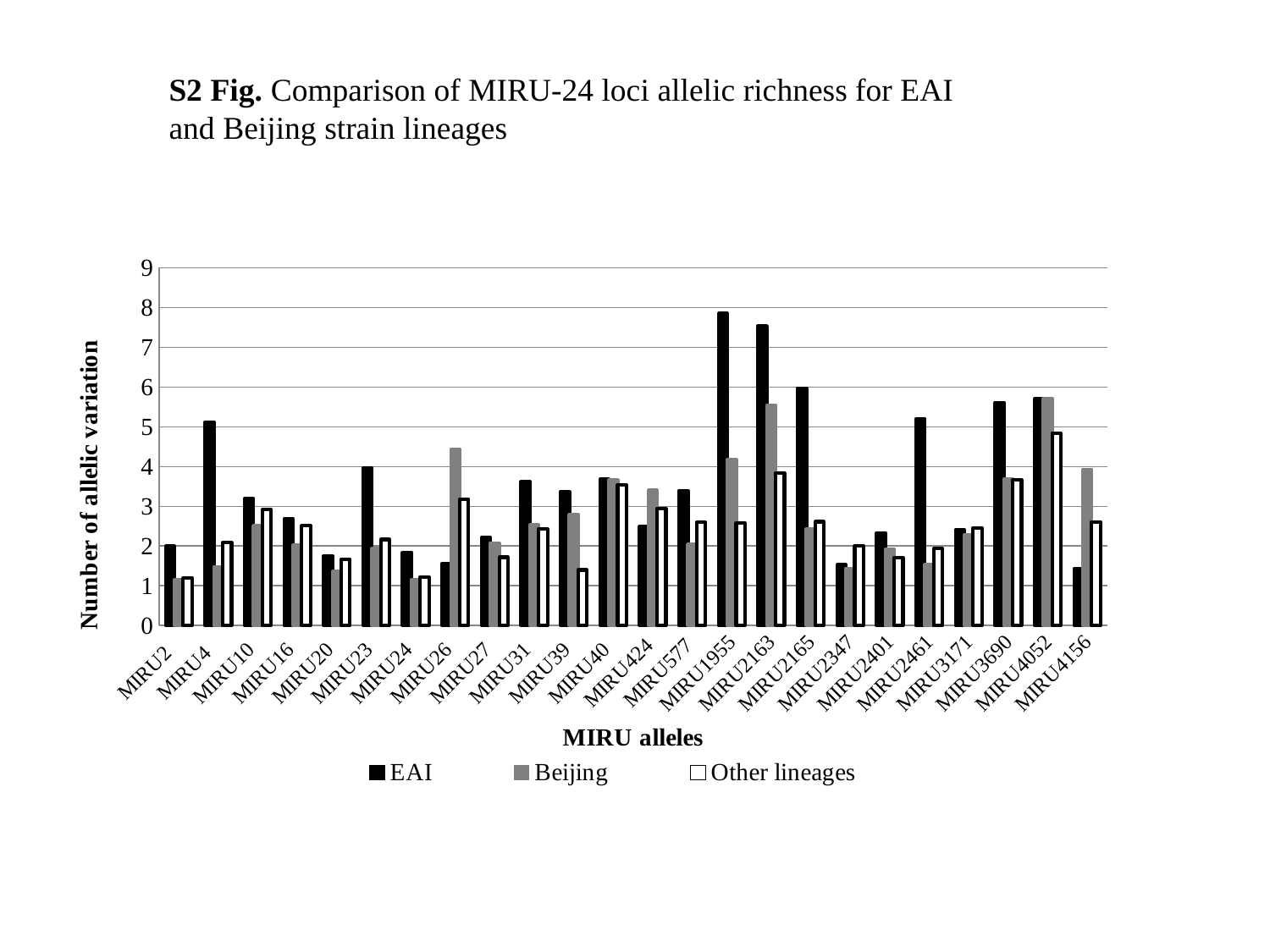

S2 Fig. Comparison of MIRU-24 loci allelic richness for EAI and Beijing strain lineages
### Chart
| Category | | | |
|---|---|---|---|
| MIRU2 | 1.9936 | 1.1489 | 1.18945 |
| MIRU4 | 5.120499999999994 | 1.4782 | 2.0893 |
| MIRU10 | 3.1964 | 2.5024 | 2.9197375 |
| MIRU16 | 2.6878 | 2.0154 | 2.50831875 |
| MIRU20 | 1.7527 | 1.356 | 1.6629375 |
| MIRU23 | 3.9655 | 1.9337 | 2.16256875 |
| MIRU24 | 1.8372 | 1.1447 | 1.21851875 |
| MIRU26 | 1.5609 | 4.427799999999999 | 3.17851875 |
| MIRU27 | 2.2146 | 2.075899999999999 | 1.715125 |
| MIRU31 | 3.6175 | 2.5306 | 2.42375 |
| MIRU39 | 3.3738 | 2.7952 | 1.39554375 |
| MIRU40 | 3.6872 | 3.6608 | 3.5399875 |
| MIRU424 | 2.4912 | 3.399799999999999 | 2.94315625 |
| MIRU577 | 3.3967 | 2.0365 | 2.60293125 |
| MIRU1955 | 7.8473 | 4.174899999999996 | 2.57961875 |
| MIRU2163 | 7.5361 | 5.5371 | 3.83470625 |
| MIRU2165 | 5.9698 | 2.4242 | 2.611499999999999 |
| MIRU2347 | 1.5235 | 1.4373 | 1.99740625 |
| MIRU2401 | 2.3287 | 1.911899999999999 | 1.7105875 |
| MIRU2461 | 5.2018 | 1.5364 | 1.9341375 |
| MIRU3171 | 2.4092 | 2.2793 | 2.45879375 |
| MIRU3690 | 5.610199999999994 | 3.6845 | 3.6617875 |
| MIRU4052 | 5.6968 | 5.7112 | 4.843524999999984 |
| MIRU4156 | 1.4301 | 3.926899999999998 | 2.60884375 |
