# Supplementary material for: Whole Genome Sequencing Demonstrates Limited Transmission within Identified Mycobacterium tuberculosis Clusters in New South Wales, Australia
Source: PLoS One. 2016 Oct 13;11(10):e0163612. doi: 10.1371/journal.pone.0163612 (PMC5063377; doi:10.1371/journal.pone.0163612)

## Slide 1
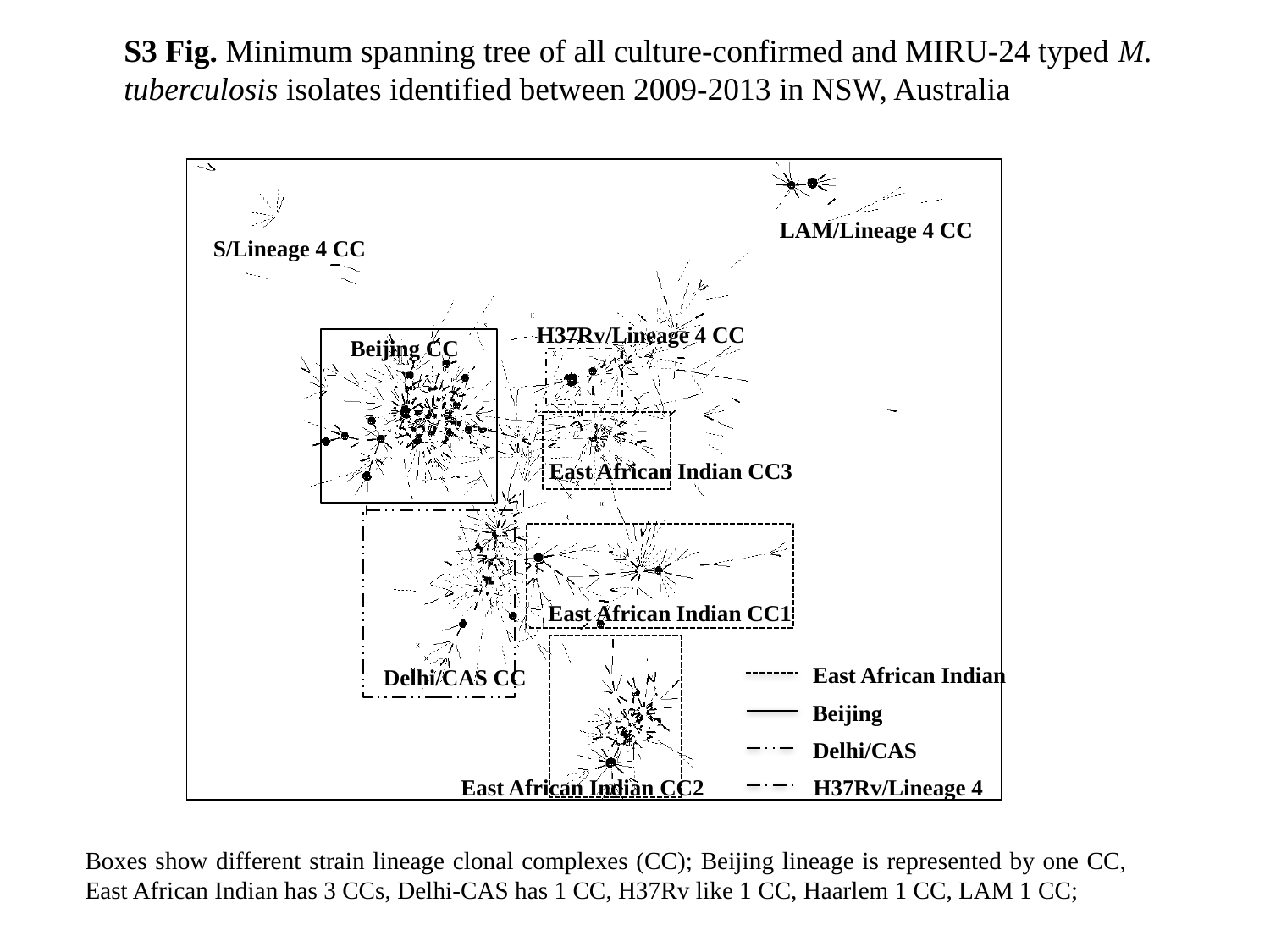

S3 Fig. Minimum spanning tree of all culture-confirmed and MIRU-24 typed M. tuberculosis isolates identified between 2009-2013 in NSW, Australia
LAM/Lineage 4 CC
S/Lineage 4 CC
H37Rv/Lineage 4 CC
Beijing CC
East African Indian CC1
East African Indian CC3
Delhi/CAS CC
East African Indian
Beijing
Delhi/CAS
H37Rv/Lineage 4
East African Indian CC2
Boxes show different strain lineage clonal complexes (CC); Beijing lineage is represented by one CC, East African Indian has 3 CCs, Delhi-CAS has 1 CC, H37Rv like 1 CC, Haarlem 1 CC, LAM 1 CC;

## Slide 2
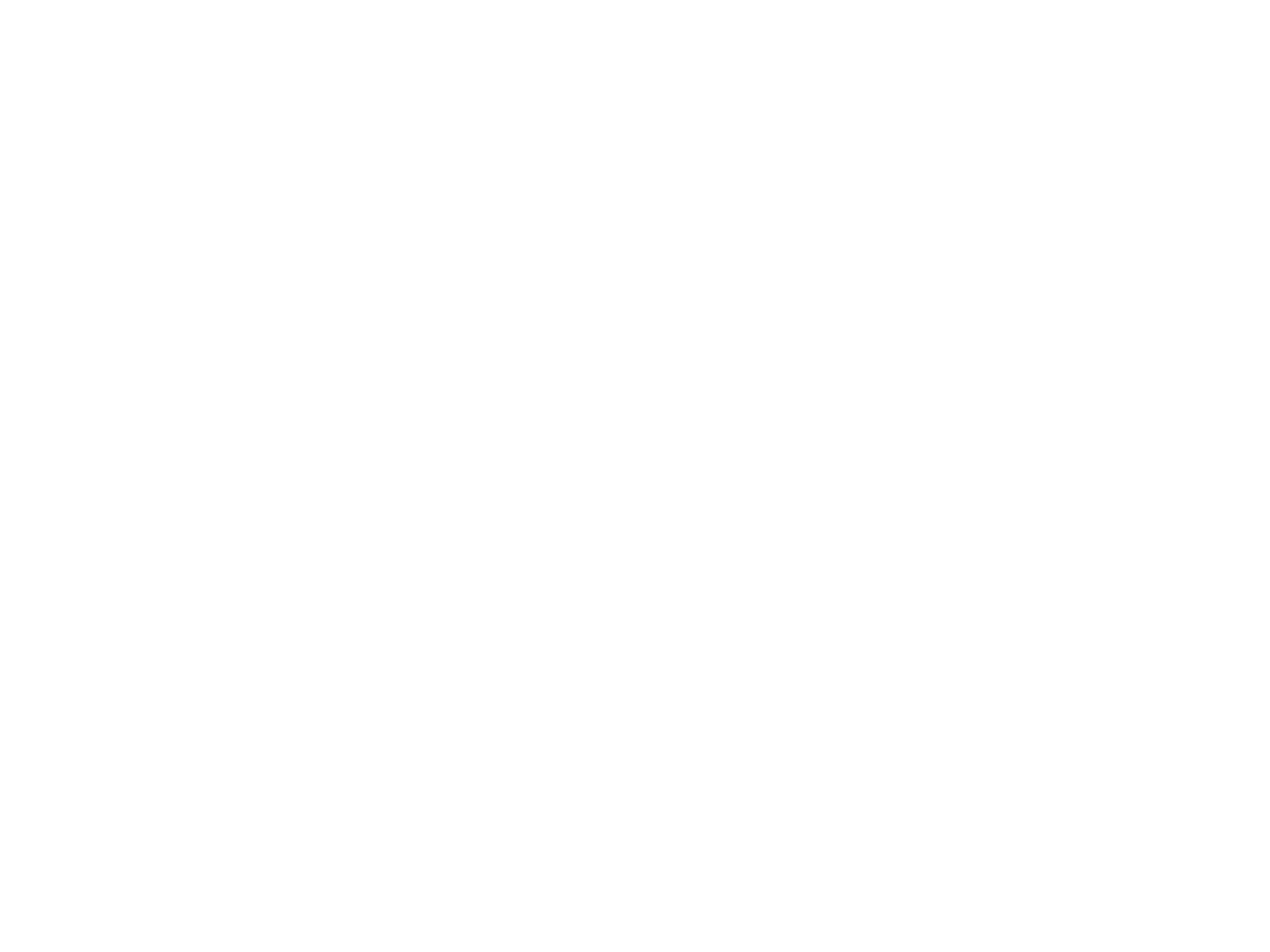

Supplement: S3 Fig — (PPTX) [file pone.0163612.s004.pptx]
